# Upcycling Roman Chamomile Hydrolate and Apple Pomace Agri-Wastes into Sustainable Cosmetic Ingredients

**DOI:** 10.3390/antiox15030380

**Published:** 2026-03-18

**Authors:** Kamil Szymczak, Agnieszka Krajewska, Małgorzata Grzyb, Iga Jodłowska, Katarzyna Mietlińska, Radosław Bonikowski

**Affiliations:** 1 Institute of Natural Products and Cosmetics, Faculty of Biotechnology and Food Sciences, Lodz University of Technology, Stefanowskiego 2/22, 90-537 Lodz, Poland; agnieszka.krajewska@p.lodz.pl (A.K.); malgorzata.grzyb@p.lodz.pl (M.G.); katarzyna.mietlinska.500@guest.p.lodz.pl (K.M.), radoslaw.bonikowski@p.lodz.pl (R.B.); 2 Institute of Molecular and Industrial Biotechnology, Faculty of Biotechnology and Food Sciences, Lodz University of Technology, Stefanowskiego 2/22, 90-537 Lodz, Poland; iga.jodlowska@p.lodz.pl

**Keywords:** cosmetics, cream, apple extract, chamomile hydrolate, polyphenols, wrinkles

## Abstract

The aim of this study was to evaluate the potential of selected agri-food by-products—apple pomace extract from *Malus domestica* cv. ‘Grochówka’ and Roman chamomile (*Chamaemelum nobile* L.) hydrolate—as functional, sustainable ingredients for cosmetic applications. The work focused on their chemical composition, biological activity, formulation performance, and in vivo effects on skin condition. Volatile compounds, phenolic acids, and triterpenoids were analyzed by GC–MS, while total phenolic content, antioxidant capacity, and enzyme inhibitory activity were evaluated in vitro. An oil-in-water emulsion containing the by-products was formulated and, in a 14-day split-face study, assessed for its effects on skin hydration, elasticity, inflammation, sensitivity, pore visibility, and melanin index. Biochemical analyses have shown that chamomile hydrolate is characterized by very low antioxidant activity (DPPH 5.0 ± 1.25%, FRAP 0%) and weak protease inhibition (9.70 ± 1.84%). In contrast, apple extract contained a significant amount of polyphenols (23.94 ± 0.3 mg GAE/g) and showed strong antioxidant properties (DPPH 79.4 ± 2.12%, FRAP 70.56 ± 2.23%; IC_50_ = 21.5 ± 0.196 mg/mL), which confirms the dominant role of phenolic compounds in its biological activity. This extract also demonstrated significant protease inhibition (60.88 ± 2.35%; IC_50_ = 15.02 ± 0.47 mg/mL), while its lipase inhibition activity was moderate (10%), which may be beneficial from a cosmetic perspective. The obtained results indicate that apple extract is a valuable raw material with multifaceted biological potential. Overall, the results demonstrate that apple pomace extract and chamomile hydrolate can be effectively valorized as bioactive cosmetic ingredients, supporting both skin health benefits and circular economy principles in sustainable cosmetic formulation.

## 1. Introduction

Over the last few years, the cosmetics market has undergone significant changes, driven by increasing consumer demand for product quality, safety, and ethical responsibility. The growth of environmental awareness has shifted consumers’ focus towards selecting natural and organic cosmetics, with a preference for plant-derived ingredients and biodegradable packaging. Consumers now value transparency from brands; they expect clear information about the ingredients’ origin and manufacturing processes [1]. Technological innovation, such as personalized formulations, has gained significant popularity, enabling the design of personalized cosmetics, especially suited to individual skin needs [2]. There is also a strong focus on products that provide long-term health benefits, such as anti-ageing skincare and formulations that improve skin condition at the cellular level. At the same time, there is an expanding interest in multifunctional products that combine nourishment with protection against external stressors, such as air pollution [3]. Furthermore, there is a growing trend of minimalism in formulation design. Consumers have started to favour products with short ingredient lists, as they prefer those that offer simplicity and efficacy [1]. In response to these changes in the market, companies are rethinking their strategies to emphasize sustainability and circularity [4]. The use of industrial by-products as raw materials not only supports the principles of a circular economy but also meets the expectations of modern consumers, seeking responsible production and consumption practices.

A prime example of food-processing waste is apple pomace, the material left after pressing the fruits of *Malus domestica* Borkh. for juice and cider [5]. It is often underutilized, despite limited applications such as animal feed, composting, or low-value processing. In this context, its transformation into a functional cosmetic ingredient with demonstrated biological activity represents a clear increase in added value—upcycling—compared to its conventional uses. This by-product is rich in health-promoting phytochemicals, especially polyphenols, renowned for their strong antioxidant capacity [6]. Owing to this abundance of beneficial compounds, apple-derived residues can be transformed into extracts with significant value for dermatological and cosmetic applications. These extracts help shield the skin from oxidative stress by neutralizing free radicals, which in turn supports the maintenance of youthful, healthy-looking skin. In addition to their antioxidative effects, apple polyphenols also exhibit notable anti-inflammatory activity, making them particularly suitable for use in formulations such as creams, masks, or serums targeting sensitive, irritation-prone, or mature skin [7,8]. Moreover, apple pomace retains substantial levels of triterpenoic acids, including ursolic acid, which is widely recognized for its pronounced anti-wrinkle and skin-firming properties [9].

Roman chamomile (*Chamaemelum nobile* L.) hydrolates are highly appreciated in cosmetics for their numerous biological activities, which are beneficial to skin health [10]. They exhibit soothing and anti-inflammatory effects, making them well-suited for formulations targeting sensitive, irritated, or allergy-prone skin [11]. As they are rich in flavonoids and other bioactive compounds, chamomile hydrolates also demonstrate strong antioxidant properties [12], protecting the skin from oxidative stress and premature ageing [13]. Their antibacterial potential further supports applications in products for acne-prone skin. Additionally, their moisturising and regenerating capabilities help maintain skin hydration [14]. Roman chamomile hydrolates also exhibit astringent properties, which improve skin elasticity and reduce the visibility of pores. Moreover, they support skin regeneration by accelerating the healing of minor wounds, burns, and irritations. Due to their ability to support cellular renewal, they are frequently incorporated into anti-wrinkle and anti-ageing cosmetic formulations [15]. The use of chamomile hydrolate, a by-product of essential oil distillation, aligns with the concept of more comprehensive utilization of plant raw materials and reducing post-production waste streams. Furthermore, replacing process water with hydrolate eliminates the need to use water of drinking water quality standards, thus contributing to the rationalization of water resource use in the cosmetic manufacturing process [16,17]. This contributes to the concept of a circular economy in multiple ways.

This study aimed to characterize two specific agri-waste by-products: hydrolate from roman chamomile (*Chamaemelum nobile* L.), obtained as a by-product of essential-oil distillation, and pomace from apple (*Malus domestica* Borkh.)—a major by-product of apple juice production—in terms of bioactive properties. As a second stage, the aim was to formulate and characterize a novel cosmetic emulsion incorporating those ingredients and evaluate its effectiveness in improving skin properties. This work also aimed to demonstrate the value of agri-waste products as functional ingredients in sustainable skin-care formulations, which is in line with circular-economy principles.

## 2. Materials and Methods

The hydrolate was obtained from Połczyno Biofarm company (Połczyno, Poland) as a product of steam distillation. Until the cosmetic formulation was prepared, it was stored refrigerated (4 °C) in glass bottles with tight caps and teflon septa, and for no longer than two weeks from the date of production. The apple pomace was obtained from the remains of juicing the native ‘Grochówka’ cultivar. Immediately after production, it was dried in warm air at 50 °C for approximately 12 h. After this time, it was put into glass jars with Teflon septa and frozen at −80 °C until the beginning of the study. To obtain the apple extract, 20 g of the raw material was placed in a Soxhlet thimble and then extracted for two hours using 200 mL of 96% ethyl alcohol. After the process was completed, the extract was evaporated to dryness using a rotary evaporator (Buchi R-215 Rotary Evaporator System, BÜCHI Labortechnik AG, Flawil, Switzerland) and then left overnight in an open flask at room temperature to remove any residual alcohol. To produce the cosmetic formulation, the dry residue was dissolved in a glycerin-water mixture (80:20) to obtain a concentration of 120 mg/mL.

### 2.1. GC-MS Analysis of Volatile Organic Compounds

The composition of volatile compounds not only determines the olfactory characteristics of the raw material incorporated into a cosmetic formulation, but also provides critical information regarding the presence or absence of potentially allergenic constituents. Both the hydrolate and the apple glycerin-water extract were analyzed using HeadSpace Solid Phase Microextraction (HS-SPME) and gas chromatography mass spectrometry (GC-MS). Briefly, 5 mL of the sample was transferred to 20 mL SPME vials and tightly sealed with a metal cap and a Teflon-rubber septum. Analyses were performed using DVB/CAR/PDMS fiber coatings obtained from Supelco (Bellefonte, PA, USA). The operating conditions were as follows: the samples were incubated and extracted at 30 °C, with incubation lasting 15 min and extraction 60 min. The adsorbed analytes were then thermally desorbed for 10 min at 250 °C using the splitless mode. GC-MS analyses were conducted on a Thermo Scientific Trace GC Ultra system coupled to a DSQ II mass spectrometer (Thermo Scientific S.p.A., Milan, Italy). Separation of the compounds was achieved on an RTX-1 capillary column (30 m × 0.25 mm, film thickness 0.25 µm) supplied by Restek (Bellefonte, PA, USA). Helium (purity 5.0) was used as the carrier gas at a constant flow of 0.5 mL/min. The oven temperature program initiated at 50 °C and was held for 3 min, then increased at a rate of 4 °C/min up to 280 °C, where it was held for an additional 10 min. The total run time was 70.5 min. The injector temperature was set at 250 °C. Mass spectra were recorded over an m/z range of 33–400 amu. The ion source temperature was maintained at 200 °C, and the transfer line was kept at 280 °C. All analyses were performed in triplicate.

### 2.2. GC-MS Analysis of Phenolic Acids

The determination of phenolic compound content is essential, as these compounds play a key role in the biological activity of plant-derived raw materials. Phenolic constituents are closely associated with antioxidant, protective, and anti-aging effects, which are highly relevant for cosmetic applications. Moreover, their quantification enables the correlation of chemical composition with functional properties and supports the scientific justification of ingredient efficacy. Apple extract was analyzed using GCxGC-MS according to the previously described method [6]. Initially, 0.2 g of the dried extract was mixed with 5 mL of a 1 M methanolic NaOH solution and heated. Subsequently, an aqueous 2 M HCl solution was added to the reaction mixture to achieve a final hydrochloric acid concentration of approximately 0.5 M in the flask. Then, methanol was evaporated on a rotary evaporator, and 3 mL of ethyl acetate was added and well shaken. For analysis, 0.2 mL of the ethyl acetate fraction was transferred to a 1.5 mL glass vial and evaporated to dryness under a nitrogen stream. The residue was derivatized with 0.2 mL of BSTFA + TMCS (99:1) at 80 °C for 2 h and subsequently analyzed by the LECO Pegasus 4D system equipped with an Agilent 7890A gas chromatograph coupled to a time-of-flight mass spectrometer (LECO, Tychy, Poland). Compound separation was achieved using a column set consisting of a BPX5 column (30 m × 0.25 mm × 0.25 μm; SGE) as the first-dimension column and a BPX50 column (2 m × 0.10 mm × 0.10 μm; SGE) as the second-dimension column. Helium was used as the carrier gas at a constant flow rate of 1.0 mL/min. The oven temperature program started at 50 °C (held for 1 min), followed by an increase of 6 °C/min to 330 °C and a final hold of 10 min, giving a total analysis time of 58 min. The second-dimension oven temperature was maintained 5 °C higher than that of the first dimension. The injector temperature was set at 200 °C. The mass spectrometer operated in electron impact mode at −70 eV, with a scan range of m/z 33–750 amu. The ion source and transfer line temperatures were set to 200 °C and 250 °C, respectively. Quantitative determination of phenolic compounds in the samples was based on a calibration curve constructed using gallic acid in concentrations of 25 mg/L, 50 mg/L, 100 mg/L, 200 mg/L, and 500 mg/L.

### 2.3. GC-MS Analysis of Triterpenoids

The evaluation of triterpenoid content, including compounds such as ursolic acid and oleanolic acid, is of particular importance due to their well-documented biological activities. These triterpenoids are known to exhibit anti-inflammatory, antioxidant, and skin-protective effects, contributing to the maintenance of skin integrity and homeostasis. Furthermore, their quantification allows for a more comprehensive characterization of the functional potential of plant-derived raw materials and supports the rational development of efficacious cosmetic formulations. Apple extract was analyzed using GC-MS with the previously described method [6]. Here, 0.1 g of dry extract was transferred into glass vials, and 100 μL of pyridine and 100 μL of BSTFA + TMCS (99:1) were added. The GC–MS vials were tightly sealed and heated on a hotplate at 80 °C for 2 h. After derivatization, the samples were kept in a shaded place at room temperature for the next 24 h. Prior to analysis, the samples were transferred into glass microinserts. GC–MS analyses were performed using a LECO Pegasus 4D system equipped with an Agilent 7890A gas chromatograph coupled to a time-of-flight mass spectrometer (LECO, Tychy, Poland). Compound separation was carried out on a BPX5 column (30 m × 0.25 mm × 0.25 μm; SGE). Helium was used as the carrier gas at a flow rate of 1.5 mL/min. The oven temperature program started at 250 °C (held for 1 min), followed by an increase of 15 °C/min to 320 °C and a final hold of 15 min, resulting in a total analysis time of 21 min. The injector temperature was set at 250 °C. The mass spectrometer operated in electron impact mode at −70 eV, with a scan range of m/z 33–750 amu. The ion source and transfer line temperatures were set to 200 °C and 250 °C, respectively. Quantitative determination of triterpenoids in the samples was based on a calibration curve constructed using ursolic acid (in concentrations of 1 mg/L, 2 mg/L, 5 mg/L, 10 mg/L, and 20 mg/L) and oleanolic acid (in concentrations of 0.1 mg/L, 0.5 mg/L, 1 mg/L, 2 mg/L, and 5 mg/L).

### 2.4. Antioxidant Potential

The assessment of antioxidant potential is essential, as it provides key information on the ability of raw materials to neutralize reactive oxygen species, which play a significant role in skin aging and inflammatory processes. Such analyses allow for the evaluation of the functional efficacy of cosmetic ingredients and their contribution to skin protection. Determination of antioxidant potential was carried out using a well-established method using the 2,2-diphenyl-1-picrylhydrazyl (DPPH) reagent [6], modified for faster, more efficient, and solvent-saving assays on 96-well plates. Specifically, 50 µL of the tested sample was transferred to a well on a 96-well plate, and 150 µL of 200 µM DPPH∙ reagent was added. Three replicates were performed for each sample. After preparing the samples, the plate was covered to prevent oxygen access and incubated in the dark for 30 min. Absorbance was measured at a wavelength of 517 nm in a Multiscan GO (ThermoFisher Scientific, Waltham, MA, USA). The results were converted from the standard curve to an equivalent amount of Trolox (mg TE/g) and also expressed as a percentage of free radical scavenging.

Ferric reducing/antioxidant power (FRAP) assay was performed with the modified procedure used in previous studies [18] with minor modifications. Briefly, to 50 µL of antioxidant solution, 50 µL of EtOH (96%), 250 µL of 0.2 M phosphate buffer (pH 6.6), and 250 µL of K_3_Fe(CN)_6_ solution (1%) were added; the mixture was incubated at 50 °C for 20 min. The incubated mixture was allowed to cool to room temperature, and 250 µL of TCA (10%) was added. The solution was thoroughly mixed, an aliquot of 250 µL was withdrawn, and 250 µL of water, followed by 50 µL of FeCl_3_·6H_2_O solution (0.1%), was added so that the final volume was 900 µL. The absorbance was measured after 2 min against a reagent blank at 700 nm in a Multiscan GO (ThermoFisher Scientific, Waltham, MA, USA).

### 2.5. Total Phenolic Content

Phenolic compounds are among the primary contributors to the biological activity of plant-derived raw materials. This analysis enables the estimation of the concentration of bioactive constituents responsible for antioxidant, protective, and supportive effects in cosmetic applications. The total polyphenolic content (TPC) was determined using the Folin–Ciocalteu method on a 96-well plate, as previously described [18]. Firstly, 2 µL of each extract was transferred into a well of a 96-well plate, followed by the addition of 4 µL of Folin–Ciocalteu reagent, 40 µL of 20% sodium carbonate solution, and 250 µL of distilled water. All samples were analyzed in triplicate. The prepared plate was incubated in the dark for 30 min. Subsequently, absorbance was recorded at 750 nm using a Multiscan GO microplate reader. Quantification was performed based on a calibration curve, and the results were expressed as milligrams of gallic acid equivalents per 100 g of sample (mg GAE/100 g). To determine the concentration of total polyphenols, a calibration curve was prepared using gallic acid standard in concentrations of 25 mg/L, 50 mg/L, 75 mg/L, 100 mg/L, 200 mg/L, 300 mg/L, and 400 mg/L in ethanol.

### 2.6. Proteinase Inhibitory Test

The evaluation of proteinase inhibitory activity is important, as proteinases are involved in skin aging, tissue degradation, and inflammatory processes, and their inhibition may contribute to the preservation of skin structure and function. Proteinase inhibition was assessed following a previously described method [19] with slight modifications. The reaction mixture comprised trypsin, Tris-HCl buffer (pH 7.4), and the sample solution. The reaction was carried out for 5 min at 37 °C, then 0.3% *w*/*v* of bovine serum albumin (BSA) was added, and the mixture was further incubated for 20 min. The reaction was terminated with 5% *w*/*v* trichloroacetic acid. After centrifugation (15,000 rpm, 10 °C, 10 min), 60 µL of the reaction mixture was transferred onto 96-well plates, and 120 µL of 0.5 M NaOH and 36 µL of Folin–Ciocalteu reagent (diluted 1:2 with water) were added. The absorbance was measured after 20 min of incubation in the dark against a reagent blank at 660 nm in a Multiscan GO (ThermoFisher Scientific, Waltham, MA, USA).

### 2.7. Lipase Inhibitory Test

The assessment of lipase inhibitory activity provides insight into the potential of raw materials to modulate lipid metabolism, which is relevant for maintaining skin barrier integrity and for cosmetic applications targeting sebaceous activity. The lipase inhibitory test was performed following the described method [20] using porcine pancreatic lipase (triacylglycerol acyl-hydrolase, EC 3.1.1.3; Sigma-Aldrich, St. Louis, MO, USA). The reaction mixture contained 0.33 µg/µL of lipase, 0.1 M Tris-HCl buffer with 50 mM CaCl_2_ and 1 mM EDTA, containing 100 µL of the sample solution. The prepared mixture was then incubated at 37 °C for 15 min. Thereafter, 20 µL of 10 mM p-nitrophenyl palmitate (p-NPP; Sigma-Aldrich, St. Louis, MO, USA) was added, and the mixture was incubated for an additional 15 min at the same temperature. The absorbance was measured at 400 nm using a Multiscan GO (ThermoFisher Scientific, Waltham, MA, USA).

### 2.8. Preparation of Cosmetics

Control cream (E1) and cream based on chamomile hydrolate and extract from ‘Grochówka’ pomace (E2) were prepared according to the same protocol, containing components depicted in Table 1 to obtain oil-in-water emulsions. The components of Phases A and B of both emulsions were weighed individually in separate beakers and heated up to 75 °C until complete dissolution of the solids. Then, phase A was added to phase B and homogenized at 1000 rpm using a WiseStir HS-100D mixer (Witeg Labortechnik GmbH, Wertheim, Germany) to obtain an o/w type of emulsion. Subsequently, the speed of the rotor was reduced to 500 rpm, and the emulsion was further mixed until it cooled. The components of phase C were added to the emulsion. Then, the emulsion was cooled to room temperature, and a pH measurement was performed. The whole procedure was identical for both E1 and E2.

### 2.9. Physicochemical Parameters

The color properties of the emulsions were determined using a portable colorimeter (CP100G, 3Color, Narama, Poland). The CIEL*a*b* coordinates (L*: darkness–lightness, a*: green–red axis, b*: blue–yellow axis) were determined using the standard illuminant D65, observer angle 10°, aperture size ø8 mm, specular color included (SCI) mode. The total Euclidean distance ΔE between two samples was calculated according to the following formulae: $\Delta E=\sqrt{{\left(\Delta L\right)}^{2}+{\left(\Delta a\right)}^{2}+{\left(\Delta b\right)}^{2}}$.

The pH values were measured using a CP-105 pH meter (Elmetron, Zabrze, Poland). Density was measured using a P-1 metal pycnometer (Pol-Zaf S.C., Wrocław, Poland). The viscosity of the samples was measured at 25 °C with a speed rate of 20 rpm and a shear rate of 500 s^−1^ using a rotational viscosimeter (DV1 viscosimeter, AMATEK Brookfield, Middleboro, MA, USA) with a Brookfield LV63 spindle.

### 2.10. Stability Tests

Stability tests were conducted in accordance with the developed protocol [21] on both prepared emulsions. A total of 30 g of cosmetic samples were stored for 13 weeks at room temperature (21 °C), at elevated temperatures (40 °C), and at lower temperatures (4 °C). After the specified period, the samples were evaluated with respect to color, consistency, odor, and the presence of phase separation. The obtained results were compared with those recorded prior to the stability test. Samples showing no significant changes in these parameters before and after the test were considered stable.

The stability of samples was also assessed by stress assay. Briefly, 30 g samples were stored in variable temperature conditions: 24 h at higher temperatures (40 °C) and 24 h at lower temperatures (4 °C). The test was conducted for 8 weeks. Sample stability was examined daily for color, consistency, scent, and layer separation. Samples were considered stable when these parameters showed no significant changes before and after the test.

### 2.11. Skin Biophysical Parameters

A heterogeneous group of ten female participants (25–65 years) was enrolled for the study. Ethical approval was waived in accordance with the Rules and Regulations of the Research Ethics Committee at Lodz University of Technology, and all participants provided written informed consent prior to the study.

Participants were eligible if they were free from systemic or active dermatological conditions (e.g., acne, atopic dermatitis, psoriasis, infectious skin diseases) and had not used antibiotics within the previous 3 months. Also, participants declared stable skincare habits and no history of hypersensitivity or allergies to cosmetic ingredients. The experiment employed a face-split design. Each participant applied cream E1 (basic emulsion) to the left side of the face and cream E2 (emulsion enriched with chamomile hydrolate and apple extract) to the right side, twice daily (morning and evening) for 14 days. The application of formulations to the left or right side of the face was fixed for all participants to ensure consistent application and to minimize potential cross-contamination between formulations. The physical differences between the formulations were minimal; therefore, the volunteers were not informed which formulation represented the active treatment.

During the experiment, volunteers were instructed to avoid using any additional skincare products, antimicrobial preparations, or medications that could interfere with the results. They were allowed to maintain their own skincare routines, such as skin cleansing or makeup, provided that no new cosmetic products were introduced during the study.

Skin biophysical parameters were assessed at baseline (day 0) and after 7 and 14 days of product application. All measurements were conducted under standardized environmental conditions: temperature 22 °C and relative humidity 50%. Prior to each assessment, participants cleansed and dried their faces and then rested for 15 min to allow for acclimatization. The following parameters were analyzed independently on each half of the face: moisture, elasticity, pore visibility, melanin content, inflammation, and sensitivity. Measurements were obtained using the Aram Huvis ASW-300 skin analyzer in combination with Wizard software (ver. V0.0.7) (Aram Huvis Co., Seongnam, Republic of Korea). Each parameter was calculated as the mean of three measurements taken at different points within the cheek area.

### 2.12. Statistical Analysis

Statistical analyses were conducted using GraphPad Prism version 10.4.2. All experimental data were expressed as mean ± standard deviation (SD) of at least three independent replicates. For the skin biophysical parameters, one-way analysis of variance (ANOVA) followed by Tukey’s post hoc test was used to assess the significance of differences between the parameters after application of both emulsions (E1, E2) and application times (Day 7, Day 14). Statistical significance was set at *p* < 0.05.

## 3. Results and Discussion

### 3.1. Volatile Organic Compounds (VOCs) Analysis

As shown by chromatographic analyses, the volatile compounds determined in the apple pomace extract were mainly esters (6), as well as alcohols (2), and an aldehyde (Table 2). The highest percentage shares were determined for: hexyl butyrate (37.35%), hexyl hexanoate (12.66%), and butyl hexanoate (11.03%). The analyzed profile of volatile compounds is consistent to some extent with literature data for the ‘Grochówka’ cultivar [22]. All of the identified compounds were also detected in the raw fruit, but their numbers were significantly reduced. The extract retained those components present at the highest concentrations in the raw fruit, primarily, with lower volatility compounds dominating. The remaining volatile compounds define the extract’s characteristic aromatic profile, which, although still recognizably apple-like, becomes marked by distinctly sweeter, floral, fruity, and tropical nuances of low sensory thresholds. This shift is largely driven by the predominance of hexyl butyrate, whose sweet, fruity, apple-like character imparts a rounded, mellow, and sensorially expressive foundation to the overall aroma. Complementary esters such as butyl butyrate, butyl acetate, and butyl hexanoate further enhance this profile by introducing additional layers of sweetness and tropicality, including subtle banana, pineapple, and berry notes. Simultaneously, the presence of compounds such as hexanal, hexanol, and 2-methylbutanol, associated with green, grassy, woody, and mildly alcoholic tonalities, provides a counterbalancing secondary dimension. Hexanal and hexanol reinforce the fresh, green, apple-skin facets, augmenting the perception of naturalness and freshness, while 2-methylbutanol introduces light ethereal and whiskey-like accents that add complexity without overpowering the dominant fruity signature. Together, these constituents impart a clearly developed and sensorically attractive secondary profile, enhancing the aromatic depth and contributing to the overall structural balance of the extract (Table 2).

The hydrolate contained esters (3), alcohols (2), and ketones (2) (Table 3). By far the largest percentage content was that of pinocarvone (52.82%), followed by pinocarveol (15.08%) and pinan-3-one (13.42%). The identified isobutyrates and angelate esters are compounds highly characteristic of the essential oil obtained from Roman chamomile [23]. Similarly, the main compound, pinocarvone, was identified in essential oil from this plant [12] as well as pinan-3-one [24] and pinocarveol [25].

**Table 3:** Content of major volatile compounds and their flavor analyzed in Roman chamomile hydrolate. The data of flavor comes from databases available on the following websites: leffingwell.com, thegoodscentscompany.com. R.T.—retention time; RI exp—experimental retention index; RI lit—retention index from NIST database.

Compound	R.T. [min]	RI Exp	RI Lit	% Content	Flavor	Perception Threshold [ppb]
Isobutyl isobutyrate	12.32	901	900	1.23 ± 0.02	fruity, pineapple, tropical	30
Isopentyl isobutyrate	13.57	996	996	2.48 ± 0.19	fruity, ethereal, tropical, green, grape	40
Isobutyl angelate	15.63	1049	1051	8.36 ± 0.73	herbal, green, woody	n/d *
Pinocarveol	17.97	1108	1111	15.08 ± 0.96	herbal, camphor, woody, pine	25
Pinocarvone	18.55	1123	1124	52.82 ± 2.30	herbal, minty	n/d
Pinan-3-one	19.01	1135	1134	13.42 ± 0.88	cedar, camphoreous	n/d
Borneol	19.77	1154	1152	2.13 ± 0.17	camphoreous, herbal, woody	80
		% of identified:		95.52		

* n/d—no data.

Hydrolate is characterized by a distinctly herbal-camphorate aromatic profile, determined primarily by its high pinocarvone content (Table 3). This gives the composition a cool, slightly minty tone with a distinct sharpness and freshness. A significant pinocarveol content introduces additional herbal, green, and resinous nuances, enhancing the impression of freshness and subtle pine notes. Also, pinan-3-one, on the other hand, accentuates the cool, cedar-camphorate aspects, giving the aroma greater depth, dryness, and woodiness. In the background, delicate, fruity ester notes (including isobutyl isobutyrate and isopentyl isobutyrate) are perceptible, bringing subtle notes of pineapple, grape, and tropical fruit. However, these are merely components of the composition, softening its dominant character and lending it a touch of sweetness and an ethereal lightness. Overall, the scent can be described as intensely herbal, cool, resinous, and camphorous, with distinct woody undertones and a soft, fruity, and tropical aura in the background.

The use of apple extract and chamomile hydrolate, rich in natural fragrance compounds with high sensory acceptability by consumers, allows for a pleasant fragrance profile of the product without the need to introduce additional perfumes into the cosmetic formulation.

An important aspect of cosmetic product safety assessment is the allergen content of raw materials used in the formulation. According to current regulations (Regulation (EC) 1223/2009 [26] and its amendment, Regulation (EU) 2023/1545 [27]), all substances classified as fragrance allergens must be listed in the ingredients list if their concentration exceeds 0.001% in leave-on products and 0.01% in rinse-off products. This amendment expanded the list of allergens from 26 to over 80 substances, also including numerous terpenes naturally present in essential oils.

Chamomile essential oil contains several compounds considered allergenic, including linalool, α-pinene, β-pinene, and other monoterpenes typical of plant raw materials. In the case of our chamomile hydrolate, none of the compounds included in the current list of fragrance allergens were detected. Consequently, the hydrolate can be considered a raw material with lower allergenic potential, although reactions to trace amounts of plant ingredients are still possible in individuals with particularly sensitive skin.

### 3.2. Phenolic Acids Content

The GC–MS analysis allowed for the identification and quantification of five phenolic acids in their trimethylsilyl derivatives (Table 4). Phloretic acid was the most abundant phenolic constituent, reaching 5.23 ± 0.07 mg/g, while the remaining acids were present at comparable but lower levels, ranging from 0.81 to 1.35 mg/g. The total content of the quantified phenolic acids amounted to 9.23 ± 0.21 mg/g, indicating a visible contribution of these compounds to the overall phenolic profile of the extract. The results are consistent with the previously presented studies, which showed that the main phenolic acid in apple peel was phloretic acid, and the share of the rest of the acids was lower [6].

### 3.3. Triterpenoids Content

The GC–MS analysis enabled the identification and quantification of six triterpenoid compounds in their trimethylsilyl derivatives (Table 5). Among the identified triterpenoids, ursolic acid was the predominant compound, reaching 23.35 ± 0.11 mg/g, followed by oleanolic acid at 6.05 ± 0.07 mg/g. The remaining compounds were present at lower concentrations, ranging from 0.04 to 2.63 mg/g. The total content of quantified triterpenoids amounted to 34.22 ± 0.23 mg/g, highlighting the substantial contribution of these bioactive compounds to the overall triterpenoid profile of the extract. The results are consistent with the previously presented studies, which showed that the main triterpenoic acid in apple peel was ursolic acid, and the share of the rest of the triterpenoids was much lower [6].

### 3.4. Total Phenolic Content and Antioxidant Potential

The total phenolic content was measured using the Folin–Ciocâlteu assay, which is a technique used to detect the presence of hydroxyl groups in phenolic compounds. In the case of the chamomile hydrolate, analysed in this study, no polyphenols were detected. Furthermore, the polyphenol concentration in the ‘Grochówka’ extract was found to be 2.39 ± 0.30 mg GAE/g extract. Our previous research shows that the value of TPC for the ‘Grochówka’ cultivar was 214.2 mg GAE/100 g of extract, and the average TPC in extracts from the skins of other apple varieties was 173.2 mg GAE/100 g of extract [6]. These results demonstrate a strong correlation with the values obtained from studies conducted to date. The elevated TPC value is attributable to the high content of gallic and p-coumaric acids, as well as hyperoside and quercetin glycosides, in the skin of the ‘Grochówka’ apple cultivar [6]. Literature data are very divergent and may differ by up to several orders of magnitude depending on the variety, part of the fruit, analysis method, and the unit in which the results are expressed (equivalent of the standard substance). Research on apple pomace extracts shows values up to even 142.80 ± 26.61 mg GAE/g extract [28]. A review on apple phenolic compounds has demonstrated that the TPC possibly reaches values as high as 304.66 ± 3.74 mg GAE/100 g or 13.5 gTE/100 g DW [29].

The ability of the tested extract and hydrolates to scavenge free radicals was determined using the DPPH method. The reducing power was determined using the FRAP method. The DPPH radical scavenging activity of hydrolate from chamomile is at a low level of 5.00 ± 1.25% (3.67 ± 0.04 mM Trolox), whereas the reducing power of the hydrolate determined by the FRAP method is 0.0%. In the case of ethanol extract from ‘Grochówka’, the value of radical scavenging activity was 79.40 ± 2.12% for the 50 mg/mL solution. In comparison, the standard Trolox solution has antioxidant activity at the level of 88.99 ± 5.89%. These results indicate that apple extract has strong antioxidant properties; the assigned value of IC_50_ for ‘Grochówka’ extract is 21.5 ± 0.196 mg/mL. Conversely, the reduction power, as measured by the FRAP method, indicates that 50 mg/mL apple extract exhibits 70.56% ± 2.23% reduction. Previous studies have demonstrated the strong antioxidant properties of apple peel extracts, with an average DPPH test value of 892.4 mgTE/100 g of extract [6]. Studies by Sethi et al. 2020 also confirm the high antioxidant potential of extracts from apple cultivars, with FRAP values of 50.47–192.02 µmol Trolox/g and 71.79–137.66 µmol Trolox/g in peel and cortex [30]. In contrast, in the DPPH test, the inhibition value was 2.35% for the cortex extract and 274.82% for the peel extract [30].

The DPPH method is susceptible to even the weakest or slowest-reacting free radical scavengers, which is why a small but measurable amount of antioxidant activity was observed in the chamomile hydrolate sample. At the same time, TPC analysis confirmed the absence of polyphenolic compounds, which are the main group of strong electron donors detected in the FRAP method. Consequently, the FRAP test, which requires effective electron donors, produced a zero result, consistent with the hydrolate’s chemical profile.

A comparison of the results yielded the conclusion that ‘Grochówka’ extract possesses strong antioxidant properties, attributable to the presence of significant amounts of polyphenols and their high chemical activity. The observed consistency between the TPC, DPPH, and FRAP values indicates that phenolic compounds play a dominant role in shaping the antioxidant profile of the analysed extract, and the extract itself can be considered a valuable material with biological potential. The correlation between high FRAP activity and intense DPPH scavenging emphasizes that the extract contains compounds with a multidirectional antioxidant mechanism of action.

Although chamomile hydrolates are often described in the literature as possessing strong antioxidant activity [10,12,14], our results did not confirm this assumption. In the present study, the hydrolate showed negligible radical scavenging ability (DPPH) and no reducing power (FRAP), which is consistent with the absence of detectable polyphenols. This discrepancy may be related to significant differences in the distillation process, plant chemotype, and the limited transfer of non-volatile phenolic compounds into the hydrolate phase [11,15]. Moreover, many reports refer to essential oils or alcoholic extracts, rather than hydrolates, which naturally contain much lower concentrations of antioxidant constituents. Therefore, the commonly reported antioxidant potential of chamomile should be interpreted with caution when referring specifically to hydrolates.

### 3.5. Inhibition of Protease Activity

The precise regulation of the protease-antiprotease balance, encompassing serine proteases and their physiological inhibitors such as α1-antitrypsin and secretory leukocyte protease inhibitor (SLPI), is pivotal to maintaining optimal skin homeostasis [19]. An imbalance in this system—especially pathological intensification of proteolytic activity—initiates proteolytic degradation of essential components of the extracellular matrix, including collagen and elastin fibers [31]. The resultant processes include amplification of pro-inflammatory signalling, disruption of tissue architecture, and progressive damage to skin structures. The protease inhibitory activity of chamomile hydrolate is 9.70 ± 1.84%, which is relatively low. In contrast, the inhibitory activity of the extract from ‘Grochówka’ is as high as 60.88 ± 2.35% at a concentration of 1 mg/mL. The determined IC_50_ value was at the level of 15.02 ± 0.47 mg/mL (Table 6). These results indicate that the tested apple pomace extract has relatively high protease-inhibitory properties. When compared with available literature data, the observed effect is of a similar order of magnitude. For example, extracts obtained from different parts of *Crotalaria juncea* (flower, leaf, and root) demonstrated inhibitory effects from approximately 68% to 71%, while the positive control (indomethacin) showed the highest inhibition (almost 95%) [19]. Although direct comparison should be made cautiously due to differences in plant matrix, extraction procedures, and assay conditions, the activity recorded for the ‘Grochówka’ pomace extract falls within a comparable range, supporting its potential as a source of protease-inhibitory constituents.

### 3.6. Inhibition of Lipase Activity

For healthy, non-irritated skin and its microbiome, the physiological composition of saturated and unsaturated free fatty acids plays an important role. Regulating lipolytic activity in the skin may play a significant role in maintaining the appropriate composition of free fatty acids produced and secreted by the skin. This process might reduce inflammation, which is particularly important in the care of oily and acne-prone skin as well as skin affected by atopic dermatitis. Therefore, the ability of some compounds and mixtures to inhibit lipase activity may be considered a potentially beneficial cosmetic property. However, this effect should not be too strong or non-selective, as excessive inhibition of lipolysis can lead to lipid imbalance and a weakened skin barrier [32].

The lipase inhibitory test showed that roman chamomile hydrolate was 26.31 ± 2.44%. In the case of the inhibitory properties of the ‘Grochówka’ extract at a concentration of 1 mg/mL, only a 10.02 ± 1.20% inhibition of lipase activity was achieved. In this case, the IC_50_ parameter value is 52.4 ± 0.544 mg/mL (Table 6). When compared with literature data, the observed inhibitory activity may be considered moderate. For example, solvent fractions obtained from *Polygonum cuspidatum* demonstrated markedly stronger effects at a lower concentration (0.1 mg/mL), with inhibitory activity ranging from approximately 78% to 86%, depending on the fraction, while the positive control (orlistat) reached 98.18 ± 0.18% inhibition [20].

In contrast to highly concentrated solvent fractions designed to maximize enzyme inhibition, the apple pomace extract and chamomile hydrolate evaluated in the present study represent less purified, cosmetically relevant raw materials. From a dermatological perspective, their moderate lipase-inhibitory activity may be advantageous, as it suggests a regulatory rather than strongly suppressive effect on lipolysis. Such a profile may support sebum balance without excessively disrupting physiological lipid metabolism.

In the present study, the enzyme inhibition assays were performed at 1 mg/mL, a concentration commonly used in preliminary screening of plant extracts, intended to evaluate their intrinsic bioactivity rather than to directly reflect concentrations used in final cosmetic formulations.

The inhibitory effects observed for protease and lipase may be associated with the presence of phenolic compounds and triterpenoid constituents known to occur in apple-derived materials. Apple pomace is particularly rich in polyphenols such as chlorogenic acid, catechin, epicatechin, procyanidins, and quercetin (both free and bound in the form of glycosides), which have been reported in the literature to interact with enzymatic proteins [33,34]. These compounds may form hydrogen bonds or hydrophobic interactions with amino acid residues located in or near the catalytic site of enzymes, which can partially block substrate access or induce conformational changes in the enzyme structure, leading to reduced catalytic activity [35,36].

In addition, apple pomace extract contains pentacyclic triterpenoids such as ursolic and oleanolic acids. These compounds have also been reported as inhibitors of several hydrolytic enzymes, including lipases and proteases [37,38]. Their relatively hydrophobic structure enables interactions with enzyme active sites and may interfere with substrate binding [39]. Therefore, the inhibitory activity observed in the present study is likely the result of the combined action of multiple classes of bioactive compounds naturally present in the apple pomace extract.

### 3.7. Physicochemical Parameters of Cosmetics

**Figure 1:**
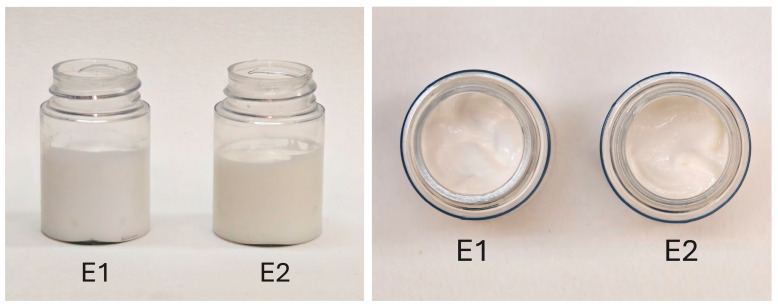
Physical appearance of the reference emulsion (E1) and the sustainable extract emulsion (E2).

As part of the research, a cosmetic emulsion formula was designed based on roman chamomile hydrolate and extract from ‘Grochówka’ pomace. The control emulsion (E1), which did not contain these ingredients, exhibited a visibly lighter color (parameter L*) compared with the formulation enriched with chamomile hydrolate and apple extract (E2, Figure 1, Table 7). This difference is expected, as the extract itself has a strong natural pigmentation. The instrumental parameters revealed a measurable difference between the formulations, with a ΔE of 8.24, indicating a significant dissimilarity between the two colours. The b* parameter, corresponding to the yellow-blue axis, further confirmed the impact of the extract on color, as E2 demonstrated a markedly higher b* value (11.67) compared with the base emulsion (5.20). This shift toward the yellow region may result from the presence of rutin and quercetin, compounds abundant in apple peels—particularly in traditional cultivars such as ‘Grochówka’ [6]—and known for their characteristic yellowish chromatic contribution due to their flavonol structure. Importantly, the yellowish tone of E2 (Figure 1) did not translate to visible staining or colour transfer on the skin.

Both formulations successfully passed stability tests, exhibiting no observable deviations in color, consistency, odor, or phase separation throughout the evaluation period. No signs of instability or other concerning changes were detected, indicating that the samples maintained their physicochemical integrity.

Physiochemical parameters such as density (0.959 g/mL for E1 and 0.967 g/mL for E2) as well as viscosity (39,180 cP for E1 and 37,980 cP for E2) are characteristic of care products in the form of emulsions. Slight differences in the obtained results may indicate that the addition of extract from ‘Grochówka’ pomace and roman chamomile hydrolate does not disturb the structure of the cosmetic/emulsion and does not affect its rheology.

Both emulsions exhibited a pH close to 5.5, which is within the physiological range of the stratum corneum, indicating that the addition of hydrolate and apple extract did not compromise the formulation’s dermatological safety and did not need to adjust the pH regulator’s concentration further. Most experts agree that the optimal pH of the topically used skin formulation should be within the range of 4–6 to assure homeostasis and barrier permeability of the skin [40]. Several recent formulation papers report that oil-in-water creams enriched with botanical extracts exhibit a similar mildly acidic pH [41,42].

The results for viscosity show no statistical differences between the two emulsions, indicating no meaningful effect of the added hydrolate and apple extract on the rheological behavior of the formulation. Although a literature search revealed no studies reporting the viscosity of creams enriched with apple extracts or chamomile hydrolates, the viscosity we observed falls within the range typical for herbal emulsions. For example, a polyherbal cream containing aloe vera gel and turmeric extract exhibited a viscosity of about 41,000 cP at 20 rpm—a value comparable to those seen in this formulation [43].

### 3.8. Assessment of Cosmetic Impact on Condition and Quality of Skin

**Figure 2:**
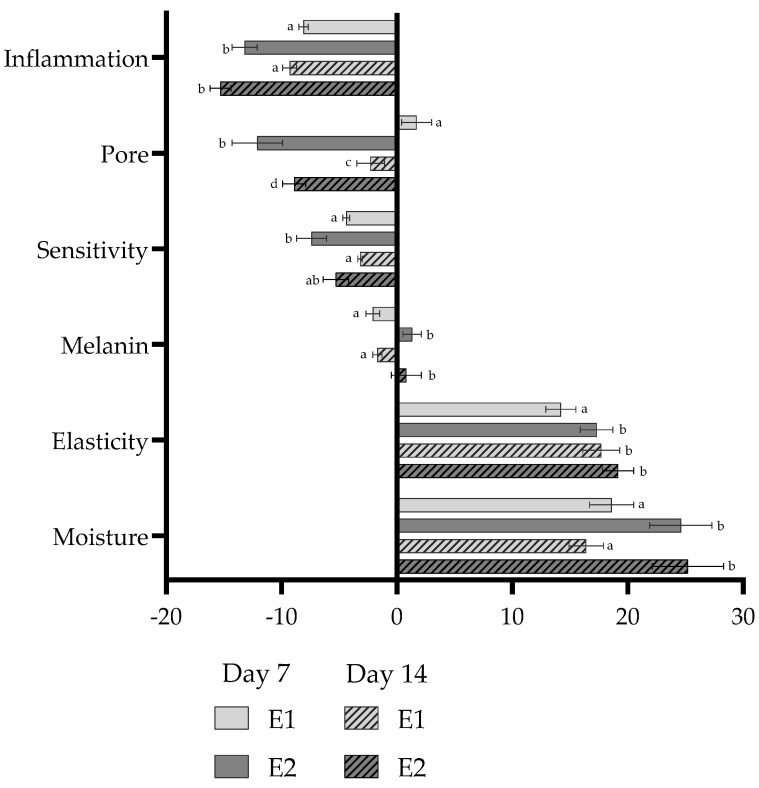
Approximate changes in skin biophysical parameters relative to baseline after applying the reference emulsion (E1) and the sustainable emulsion (E2) in a split-face test. Results are means of three replicates, with the bar denoting the standard deviation. Different lowercase letters indicate statistically significant differences (*p* ≤ 0.05) between both emulsions (E1, E2) and application times (Day 7, Day 14) within the same parameter according to Tukey’s HSD test.

Due to the split-face design, each participant served as their own control, which increases statistical sensitivity and allows detection of treatment-related changes. Improvement of skin moisture for cosmetics based on Roman chamomile hydrolate and enriched with apple pomace extract was observed up to 24.6% and 25.2% after 7 and 14 days of application, respectively, whereas control emulsions achieved a degree of hydration at the level of around 17% (Figure 2). Apple pomace extract is rich in pectins and polysaccharides, which might increase water-binding capacity and act as humectants. The results are in line with [44], where dermocosmetic creams enriched with wild apple extracts increased hydration in in vivo tests. Due to the high phenolic content and strong radical-scavenging activity of apple extract, ROS-driven lipid peroxidation in the stratum corneum can be limited, helping to preserve barrier lipids and moisturising factors. These results are comparable to those reported in an in vivo trial of creams containing wild apple extract, where a 28-day application increased hydration by 21.19 ± 7.59% to 29.60 ± 10.95% depending on extract concentration [44]. In this study, the authors observed no significant changes in transepidermal water loss or skin pH during use. Another work shows that application of a cream enriched with wild apple fruit extract assured improved skin hydration by 18.52 ± 11.51% after 14 days and 16.52 ± 9.36% after 28 days of application [45]. The similarity suggests that pectins and polysaccharides of apple pomace act as humectants and improve skin hydration; however, the final effect depends also on other constituents of the emulsion.

Inflammation in skin was markedly reduced in both emulsions, with E2 being more effective (−13.2% vs. −8.1% for control emulsion in day 7 and −15.3% vs. −9.3% for control emulsion in day 14). Chamomile is known for its ability to inhibit COX-2 [46] and suppress pro-inflammatory cytokines [47], which might have led to reduced skin microirritation. In this study, it was also proven that chamomile hydrolate presents LOX inhibition, and so, it limits peroxidation of sebum and formation of pro-inflammatory lipid mediators around follicles, which is essential in comedogenesis and acne [48]. It was demonstrated before that extracts derived from *Malus domestica* cv. ‘Grochówka’ has proapoptotic and cytoprotective properties in human-originated Caco-2 and HepG2 cell lines, further supporting the gathered results [6].

Similarly, skin sensitivity decreased with both formulations, although the reduction was more pronounced with E2. On day 7, sensitivity dropped by 7.4% for E2 compared to 4.4% for E1, and on day 14 by 5.3% vs. 3.2%, respectively. Chamomile is widely recognized for its soothing properties, particularly due to the presence of α-bisabolol and chamazulene—compounds that inhibit the production of prostaglandins and leukotrienes [49]. The results demonstrate that even in hydrolate form, chamomile retains measurable anti-irritant activity.

Both emulsions improved skin elasticity, with increases ranging from 14 to 19% across the study period. The enriched formulation showed better performance on day 7 (+17.3% vs. +14.2% for E1), with no statistically significant differences between the emulsions after another 7 days.

The melanin index decreased with E1 (−2.1 at day 7 and −1.7 at day 14) but showed slight increases with E2 (+1.3 and +0.8). There are studies showing that ethanol extracts from apple flesh and peel exhibit a whitening effect in vitro by decreasing melanin synthesis in B16/F10 melanoma cells [50]; however, this effect is strictly concentration-dependent. The lightening effect of apple extracts could require several weeks of continuous use. In the two-week trial, the sustainable formulation did not reduce melanin, and the slight increase observed may reflect short treatment duration. Longer studies are necessary to determine whether apple extract-enriched emulsion may cause visible depigmentation.

When it comes to pore size, emulsion E1 caused minimal or inconsistent changes (+1.7% on day 7; −2.3% on day 14) while E2 significantly reduced pore visibility by 12.1% and 8.9% at the same timepoints. Apples are rich in tannins [51,52,53]—compounds containing multiple hydroxyl groups that form protein complexes in the skin, resulting in astringent activity. Gathered results suggest that apple extracts could be used in cosmetics to reduce pore size and sebum secretion.

Our research addresses the current needs of the cosmetics industry in the search for innovative, sustainable, and functional raw materials of natural origin. It demonstrates the high utility of agri-food industry by-products as valuable ingredients with significant biological potential, supporting the principles of a circular economy. The article also highlights the potential for reducing waste and the consumption of primary resources in cosmetics production processes. The presented results constitute a valuable scientific and practical contribution, responding to the growing expectations of environmentally conscious consumers and producers. However, these findings should be interpreted with caution due to the exploratory nature of the study, the relatively small sample size (n = 10), and the short duration of treatment. Larger and longer-term clinical studies are required to confirm the observed effects.

## 4. Conclusions

The presented research is merely an example that valuable, health-promoting bioactive compounds do not necessarily require the search for new, previously unknown, or exotic raw materials. On the contrary, they can be obtained from very common products, even post-industrial waste. At the same time, their high bioactivity, effectiveness, and simple composition make them competitive with other products on the market. Replacing pure water with hydrolate aligns with the idea of a circular economy, and due to the presence of volatile organic compounds, there is no need for additional fragrances. The cream is characterized by a mild, subtle fragrance profile, which initially reveals delicate, floral–fruity notes, then gradually develops upon contact with the skin. Herbal, camphor, resinous, and woody notes emerge from terpene compounds, giving the composition depth, complexity, and longevity. Our cream, formulated with chamomile hydrolate and enriched with apple pomace extract, demonstrated measurable antioxidant activity and showed potential effects related to skin hydration and lipogenesis regulation under the conditions of the conducted assays. Compounds present in the apple extract added to the cream visibly improve skin hydration and elasticity and reduce the appearance of pores due to the presence of polysaccharides, pectins, and tannins. It also demonstrated anti-inflammatory and soothing potential in the applied experimental models, with observable effects on micro-irritation parameters and lipid peroxidation levels, which may support skin barrier function. Moreover, the available literature indicates that sustained reduction in oxidative stress and inflammation may contribute to long-term anti-aging effects. However, confirmation of such outcomes requires extended and well-controlled in vivo studies. Although no clear effect on discoloration has been demonstrated in the short term, regular use may bring long-term benefits in terms of improving the condition and balance of the skin.

## Figures and Tables

**Table 1:** Formulations of cosmetic emulsions.

Component	Concentration (%, *w*/*w*)	
	E1	E2
Phase A		
Purified Water	up to 100	
*Chamaemelum nobile* (chamomile) Flower Water		up to 100
Phase B		
Cetearyl olivate and Stearyl olivate	6.00	
*Brassica campestris* (Rapeseed) Seed Oil	15.00	
Phase C		
D-panthenol	1.50	
Tocopherol	2.00	
*Malus domestica* cv. Grochówka Extract		0.36
Glycerine	2.40	2.04
DHA-BA (Dehydroacetic Acid, Benzyl Alcohol, Water)	0.8	

**Table 2:** Content of major volatile compounds and their flavor analyzed in the extract from ‘Grochówka’ pomace. The data of flavor comes from databases available on the following websites: leffingwell.com, thegoodscentscompany.com. R.T.—retention time; RI exp—experimental retention index; RI lit—retention index from NIST database.

Compound	R.T. [min]	RI Exp	RI Lit	% Content	Flavor	Perception Threshold [ppb]
Methyl butyrate	4.27	707	708	9.41 ± 0.09	fruity, apple, sweet, banana	60–76
2-Methylbutanol	4.35	711	710	6.79 ± 0.08	ethereal, alcoholic, whiskey	250–300
Hexanal	5.52	762	766	4.91 ± 0.10	green, woody, apple, grassy, citrus	4.5–5
Butyl acetate	6.85	814	813	1.78 ± 0.01	sweet, banana, fruity, tropical	2–4.3
Hexanol	10.86	853	852	3.13 ± 0.05	green, fruity, apple	2500
Butyl butyrate	12.56	971	969	4.20 ± 0.06	sweet, fruity, tropical, cherry	100
Butyl hexanoate	20.50	1173	1173	11.03 ± 0.21	fruity, pineapple, berry, apple	250
Hexyl butyrate	21.07	1188	1188	37.35 ± 1.15	sweet, fruity, apple	250
Hexyl hexanoate	27.90	1375	1374	12.66 ± 1.04	sweet, fruity, green, tropical	n/d *
		% of identified:		91.26		

* n/d—no data.

**Table 4:** Content of phenolic acids analyzed in the extract from ‘Grochówka’ extract. R.T.—retention time; RI exp—experimental retention index; RI lit—retention index from NIST database.

Compound	R.T. [min]	RI Exp	RI Lit	Content [mg/g]
4-Hydroxybenzoic acid [2TMS]	16.54	1633	1635	0.98 ± 0.02
Phloretic acid [2TMS]	18.33	1767	1762	5.23 ± 0.07
Protocatechuic acid [3TMS]	19.91	1838	1834	1.35 ± 0.01
p-Coumaric acid [2TMS]	22.20	1946	1942	0.86 ± 0.02
Caffeic acid [3TMS]	26.11	2161	2158	0.81 ± 0.04
			Total	9.23 ± 0.21

**Table 5:** Content of triterpenoids analyzed in the extract from ‘Grochówka’ extract. R.T.—retention time; RI exp—experimental retention index; RI lit—retention index from NIST database; n/d—no data.

Compound	R.T. [min]	RI Exp	RI Lit	Content [mg/g]
α-Amyrin [TMS]	11.68	3441	3429	1.81 ± 0.02
Betulin [2TMS]	14.05	3580	3560	0.34 ± 0.02
Oleanolic acid [2TMS]	14.17	3598	3591	6.05 ± 0.07
Betulinic acid [2TMS]	14.46	3604	3588	0.04 ± 0.00
Ursolic acid [2TMS]	15.23	3669	3657	23.35 ± 0.11
Corosolic acid [3TMS]	17.03	3782	n/d	2.63 ± 0.02
			Total	34.22 ± 0.23

**Table 6:** The inhibitory properties of roman chamomile hydrolate and ‘Grochówka’ apple extract towards the activity of lipase and protease.

Activity	IC_50_ [mg/mL]	Percent of Inhibition [%]
Roman chamomile hydrolate		
Lipase	n.d.	26.31 ± 2.44
Protease	n.d.	9.70 ± 1.84
‘Grochówka’ extract		
Lipase	52.4 ± 0.54	10.00 ± 1.20
Protease	15.02 ± 0.47	60.88 ± 2.35

n.d.—not determined.

**Table 7:** Color parameters (CIE L*a*b*) and physicochemical parameters (density, pH, and viscosity) of the reference emulsion (E1) and the sustainable emulsion (E2). Values are means of three replicates ± standard deviation. Different lowercase letters indicate statistically significant differences (*p* ≤ 0.05) between both emulsions (E1, E2) within the same parameter according to Tukey’s HSD test.

	E1	E2
L*	86.87 ± 2.13 ^a^	81.92 ± 1.64 ^b^
a*	−1.06 ± 0.07 ^a^	−2.32 ± 0.09 ^b^
b*	5.2 ± 0.14 ^a^	11.67 ± 0.68 ^b^
ΔE	8.24	
Density [g/mL]	0.959 ^a^	0.967 ^b^
pH	5.52 ^a^	5.48 ^a^
Viscosity [cP]	39,180 ± 420 ^a^	37,980 ± 512

## Data Availability

The original contributions presented in this study are included in the article. Further inquiries can be directed to the corresponding author.
